# Contemporary characteristics, outcomes and novel risk score for Takotsubo cardiomyopathy: a national inpatient sample analysis

**DOI:** 10.1136/openhrt-2024-002922

**Published:** 2024-10-07

**Authors:** Ankit Agrawal, Umesh Bhagat, Abdullah Yesilyaprak, Aqieda Bayat, Aanchal Sawhney, Aro Daniela Arockiam, Elio Haroun, Michael Faulx, Milind Y Desai, Wael Jaber, Venu Menon, Brian Griffin, Tom Kai Ming Wang

**Affiliations:** 1 Department of Cardiovascular Medicine, Heart, Vascular, and Thoracic Institute, Cleveland Clinic, Cleveland, Ohio, USA; 2 Department of Hospital Medicine, Cleveland Clinic, Cleveland, Ohio, USA; 3 Department of Internal Medicine, Wayne State University School of Medicine, Detroit, Michigan, USA; 4 Department of Internal Medicine, Crozer-Chester Medical Center, Upland, Pennsylvania, USA

**Keywords:** cardiomyopathy, acute coronary syndrome, cardiac catheterization

## Abstract

**ABSTRACT:**

**Background:**

Takotsubo cardiomyopathy (TC) is an established differential diagnosis of myocardial infarction with non-obstructive coronaries with significant interest but limited data on prognostication. We reviewed the characteristics and in-hospital outcomes and developed a novel risk score for TC.

**Methods:**

Using the National Inpatient Sample data from 2016 to 2020, we identified adult patients (≥18 years) with acute coronary syndrome (ACS) and TC. We divided the cohort into ACS with and without TC and retrieved baseline data. Multivariable regression analysis was conducted to identify factors associated with TC diagnosis and adverse outcomes, leading to the development of a risk-scoring system.

**Results:**

Among 7 219 004 adult ACS admissions, 78 214 (1.0%) were diagnosed with TC, with a mean age of 68.2 years, 64 526 (82.5%) being female and 5475 (7.0%, compared with 8.4% for other ACS) in-hospital mortality events. Factors significantly associated with TC were female sex (OR 6.78 (95% CI 6.47 to 7.09), p<0.001) and chronic heart failure (OR 1.60 (95% CI 1.54 to 1.66), p<0.001). A novel risk score was developed, including the following parameters: male sex, age >70 years, non-white race, hypertension, hyperlipidemia, history of coronary artery bypass grafting, history of percutaneous coronary intervention, cardiac arrhythmias, renal failure, cardiogenic shock and vasopressor use. The area under curves for in-hospital mortality was 0.716 in the derivation and 0.725 in the validation cohorts.

**Conclusions:**

TC remains a high-risk diagnosis in a minority of ACS cases, with mortality rates similar to other ACS causes. Our novel risk score offers a valuable tool for risk stratification in patients with TC, but external validation is needed to confirm its utility.

**WHAT IS ALREADY KNOWN ON THIS TOPIC**

- Takotsubo cardiomyopathy presents as an acute heart failure syndrome and is associated with considerable morbidity and mortality.

**WHAT THIS STUDY ADDS**

- In this nationwide study, we have designed a novel risk score that can predict in-hospital mortality associated with Takotsubo cardiomyopathy.

**HOW THIS STUDY MIGHT AFFECT RESEARCH, PRACTICE OR POLICY**

- Our novel risk score will help in risk stratification of the patients with Takotsubo cardiomyopathy, thereby aiding in optimal management.

## Introduction

 Takotsubo cardiomyopathy (TC), also known as stress-induced cardiomyopathy or broken heart syndrome, is an intriguing cardiac syndrome characterised by left ventricular (LV) systolic dysfunction, often transient and triggered by significant emotional or physical stress.^1^ The clinical, electrocardiogram (ECG), and biomarker presentation is dramatic, mimicking myocardial infarction. Therefore, coronary catheterisation is routinely performed to exclude obstructive coronary heart disease and plaque rupture before TC can be diagnosed.^2^ Several pathophysiological mechanisms have been implicated in TC, including overstimulation of the sympathetic system, microvascular and myocardial tissue metabolism abnormalities, coronary artery vasospasm^3 4^ and catecholamine surge related to emotional distress and may render the distal LV chamber selectively vulnerable to myocardial stunning.^5^ Furthermore, given the recovery these patients have within weeks of presentation, supportive care with medical management is key.^6^ Key prognostic factors to guide optimal management strategy in TC are not well-established. This study aims to assess the clinical characteristics, outcomes and prognostic predictors and develop a novel prognostic risk score in patients with TC.

## Methods

### Data source

The National Inpatient Sample Database (NIS), one of the largest databases of inpatient hospitalisation of all-age, all-payer discharges in the US non-federal hospitals managed by the Healthcare Cost and Utilisation Project (HCUP), was used in this study.^7^ The NIS dataset constitutes a stratified sample from 22 states with data from >17 million hospitalisations annually. The database includes anonymised information on patient demographics, admission status, discharge diagnosis, comorbidities, procedures and outcomes for each sampled hospitalisation. We used datasets from 2016 to 2020 using the International Classification of Diseases, tenth revision (ICD-10) diagnostic codes.

### Study characteristics and outcomes

All adults (aged ≥18 years) with a diagnosis of TC during 2016–2020 were included in the study. Diagnosis of TC was identified using ICD-10 codes (I51.81) (online supplemental table S1). The cohort was divided into two major categories: acute coronary syndrome (ACS) with TC and without TC. ACS included acute myocardial infarction (ST-segment elevation and non-ST segment elevation) and unstable angina. Patients’ baseline characteristics such as age, sex, race, hospital region, size, teaching or non-teaching hospital, comorbidities, clinical presentation and outcomes were extracted from the dataset. The patients’ clinical characteristics and in-hospital outcomes were defined using the ICD-10 diagnostic codes. HCUP classifies race into White, African American, Hispanic and Asian or Pacific Islander. Comorbidities taken into consideration were a history of coronary artery bypass grafting (CABG), percutaneous coronary intervention (PCI), previous myocardial infarction, implantable cardiac defibrillator/permanent pacemaker (ICD/PPM) implantation, stroke, hypertension, hyperlipidemia, diabetes mellitus, obesity, smoking, heart failure, cardiac arrhythmias (including heart blocks, atrial and ventricular arrhythmias), valvular heart disease, atrial fibrillation, depression and renal failure. Individuals aged <18 years were excluded from our study. Our primary outcome was all-cause in-hospital mortality. The secondary outcomes were cardiovascular mortality (defined as patients who had either life-threatening cardiac arrhythmia like ventricular fibrillation and ventricular tachycardia or ischaemic heart disease or congestive heart failure (diastolic and/or systolic) and had mortality) and complications such as acute kidney injury (AKI) and stroke.

### Statistical analysis

Data are presented as mean±standard deviation for continuous variables and proportion for categorical variables. The temporal trend of TC hospitalisations and seasonal variation of TC-related admissions and in-hospital mortality from 2016 to 2020 was determined using Pearson’s χ^2^ test and plotted using a bar graph generated in Microsoft Excel (V.2401, Redmond, Washington, USA, 2022). The trend analysis was performed taking clustering and stratification into account. A p value of <0.05 was considered statistically significant. Data were divided into patients with ACS with and without TC and compared using Student’s t-test (or Wilcoxon rank-sum test for non-normal distribution, with normality assessed by Shapiro-Wilk test) for continuous variables and χ^2^ test (or Fisher’s exact test) for categorical variables. Multivariable logistic regression analyses were performed to identify factors independently associated with TC among patients with ACS, and the predictors of all-cause in-hospital mortality, cardiovascular mortality, AKI and stroke in patients with TC, reporting odds ratios (ORs) and 95% confidence intervals (CIs). We divided the TC cohort into patients from 2016 to 2019 as a derivation cohort to develop a risk score for in-hospital mortality in TC and patients from 2020 as a validation cohort and evaluated the area under the curve (AUC) of the receiver operating characteristic curve of the risk score in both cohorts. A beta-coefficient was calculated for each variable from which the final risk score model was derived. For further validation, we performed bootstrapping. Somers’ D statistics were used to help us assess the correlation between the predicted probabilities and observed responses. Data splitting was done for model validation, where the data were split randomly into training and test datasets. The model was resampled 1000 times with replacement. We calculated the R^2^ index and the Brier score. The R^2^ index value near 1 is preferable, whereas the Brier score near 0 is desirable. A p value of <0.05 was considered statistically significant, and all tests were two-tailed. The analysis was conducted using Stata Statistical Software V.17, 2021 (StataCorp, College Station, Texas, USA). Stata’s every command and appropriate weights were used in all estimations.

## Results

### Cohort characteristics and outcomes

Among 7 219 004 adult patients hospitalised for ACS, 78 214 (1.0%) were diagnosed with TC in the NIS 2016–2020 database. The trend analysis of TC is depicted in online supplemental figure S1. Compared with 2016, a reduction in the diagnosis of TC was reported in 2017, but in the following years, a linear increase in the diagnosis of TC was observed from 2017 to 2020 (p<0.001). TC admissions were highest in summer and fall (p<0.001), but the in-hospital mortality rate was highest in winter (p=0.01), as shown in online supplemental figure S2. Mean age is 68.2±13.3 years in TC compared with 69.1±14.1 years in patients with ACS without TC (p<0.001). Females made up 82.5% of TC, significantly higher than the 42.1% of patients with ACS without TC (p<0.001). Patients with TC had a higher proportion of white (78.0% vs 58.8%) but a lower proportion of black (7.4% vs 13.6%) race than patients with ACS without TC (p<0.001). Patients with TC had a higher proportion with a history of depression and a lower proportion with a history of CABG, PCI, myocardial infarction, ICD/PPM, stroke, hypertension, dyslipidaemia, diabetes, obesity, smoking, arrhythmias, atrial fibrillation, valvular heart disease and renal failure (p<0.001).

On presentation, TC had a higher proportion of patients with cardiogenic shock (8.3% vs 4.8%) and vasopressor use but a lower proportion getting an intra-aortic balloon pump (1.7% vs 2.7%) than patients with ACS without TC (all p<0.001). In-hospital all-cause mortality occurred in 7.0% of patients with TC, lower than the 8.0% of patients with ACS without TC (p<0.001), while cardiovascular mortality was similar at 3.9% vs 4.0% (p=0.297), respectively. Patients with TC also had lower rates of AKI (24.0% vs 32.7%, p<0.001) but higher rates of in-hospital stroke (4.4% vs 3.2%, p<0.001) (table 1).

**Table 1:** Baseline characteristics of patients with ACS with and without TC

VariablesNumber of patients	ACS without TC 7 140 790; n (%)	ACS with TC78 214; n (%)	P value	Standardised mean difference
Age (years)	69.1±14.1	68.2±13.3	**<0.001**	−0.06
Females	3 006 272 (42.1)	64 526 (82.5)	**<0.001**	1.06
Race			**<0.001**	
White	4 912 863 (68.8)	61 007 (78.0)		0.22
Black	971 147 (13.6)	5788 (7.4)		−0.23
Hispanic	592 685 (8.3)	5005 (6.4)		−0.08
Asian or Pacific Islander	649 811 (9.1)	6335 (8.1)		−0.04
Hospital region			**<0.001**	
Northeast	1 456 721 (20.4)	15 173 (19.4)		−0.02
Midwest	1 570 973 (22.0)	18 380 (23.5)		0.03
South	2 677 796 (37.5)	27 062 (34.6)		−0.06
West	1 421 017 (19.9)	17 520 (22.4)		0.06
Hospital bed size			**<0.001**	
Small	1 399 594 (19.6)	13 453 (17.2)		−0.06
Medium	2 149 377 (30.1)	22 213 (28.4)		−0.03
Large	3 584 676 (50.2)	42 470 (54.3)		0.08
Teaching hospital			**<0.001**	
Rural	549 840 (7.7)	4458 (5.7)		−0.08
Urban, non-teaching	1 478 143 (20.7)	14 860 (19.0)		−0.04
Urban, teaching	5 098 524 (71.4)	58 817 (75.2)		0.08
Median household income by zip code			**<0.001**	
0–25th percentile	2 167 229 (30.3)	20 500 (26.2)		−0.09
26th–50th percentile	1 902 306 (26.6)	20 617 (26.3)		−0.005
51st–75th percentile	1 678 085 (23.5)	20 328 (25.9)		0.05
76th–100th percentile	1 393 882 (19.5)	16 769 (21.4)		0.04
Comorbidities				
Old myocardial infarction	1 006 851 (14.1)	7195 (9.2)	**<0.001**	−0.16
History of CABG	706 938 (9.9)	1564 (2.0)	**<0.001**	−0.56
History of PCI	3 463 283 (48.5)	30 972 (39.6)	**<0.001**	−0.18
History of ICD/PPM	457 010 (6.4)	2190 (2.8)	**<0.001**	−0.22
History of stroke	856 894 (12.0)	6726 (8.6)	**<0.001**	−0.11
Cardiac arrhythmias	3 056 258 (42.8)	27 375 (35.0)	**<0.001**	−0.16
Atrial fibrillation	2 020 843 (28.3)	15 408 (19.7)	**<0.001**	−0.21
Valvular heart disease	1 199 625 (16.8)	9151 (11.7)	**<0.001**	−0.15
Hypertension	5 855 447 (82.0)	52 481 (67.1)	**<0.001**	−0.31
Hyperlipidemia	3 877 448 (54.3)	35 587 (45.5)	**<0.001**	−0.17
Diabetes mellitus	2 956 287 (41.4)	19 710 (25.2)	**<0.001**	−0.37
Chronic heart failure	3 184 792 (44.6)	34 648 (44.3)	0.561	−0.005
Renal failure	2 320 756 (32.5)	11 497 (14.7)	**<0.001**	−0.50
Obesity	1 363 890 (19.1)	9855 (12.6)	**<0.001**	−0.19
Smoking	3 099 102 (43.4)	32 693 (41.8)	**<0.001**	−0.03
Depression	799 768 (11.2)	14 547 (18.6)	**<0.001**	0.19
Anxiety	821 191 (11.5)	17 754 (22.7)	**<0.001**	0.26
Opioid use	99 971 (1.4)	1877 (2.4)	**<0.001**	0.08
Cannabis use	121 393 (1.7)	1877 (2.4)	**<0.001**	0.04
Cocaine use	92 830 (1.3)	625 (0.8)	**<0.001**	−0.04
Sedatives/Hypnotics	7140 (0.1)	312 (0.4)	**<0.001**	0.03
Stimulant use	71 407 (1.0)	547 (0.7)	**0.001**	−0.02
Severe sepsis	240 644 (3.37)	2534 (3.24)	0.374	−0.007
Septic shock	449 155 (6.29)	7219 (9.23)	**<0.001**	0.10
Dialysis	292 772 (4.10)	1157 (1.48)	**<0.001**	−0.21
Acute respiratory failure	819 048 (11.47)	14 422 (18.44)	**<0.001**	0.17
Non-invasive ventilation	784 059 (10.98)	14 125 (18.06)	**<0.001**	0.02
Intubation	623 390 (8.73)	10 926 (13.97)	**<0.001**	0.15
Presentation				
Cardiac arrest	242 786 (3.4)	2581 (3.3)	0.373	−0.007
Cardiac tamponade	7140 (0.1)	78 (0.1)	0.680	0.003
Acute mitral regurgitation	321 335 (4.5)	3989 (5.1)	**0.001**	0.02
Cardiogenic shock	342 758 (4.8)	6491 (8.3)	**<0.001**	0.12
Vasopressor use	185 660 (2.6)	3363 (4.3)	**<0.001**	0.08
Balloon pump	192 801 (2.7)	1330 (1.7)	**<0.001**	−0.07
LVAD	1428 (0.02)	–	0.104	−0.01
ECMO	7140 (0.1)	78 (0.1)	0.303	−0.009
In-hospital outcomes				
All-cause mortality	599 826 (8.4)	5475 (7.0)	**<0.001**	−0.03
Cardiovascular mortality	285 631 (4.0)	3050 (3.9)	0.297	−0.008
Acute kidney injury	285 631 (32.7)	18 771 (24.0)	**<0.001**	−0.20
Stroke	228 505 (3.2)	3441 (4.4)	**<0.001**	0.02

Numbers are mean±standard deviationSD or frequency (percentage).

ACS, acute coronary syndrome; CABG, coronary artery bypass grafting; ECMO, extracorporeal membrane oxygenation; ICD/PPM, implantable cardiac defibrillator/permanent pacemakerLVAD, left ventricle assist device; PCI, percutaneous coronary intervention; SDstandard deviationTC, takotsubo cardiomyopathy

### Predictors of Takotsubo cardiomyopathy

Table 2 lists the multivariable analysis results for factors significantly associated with TC.Female sex was independently associated with TC diagnosis among ACS admissions, OR (95% CI) of 6.78 (6.47 to 7.09), p<0.001. The other factor independently associated with TC diagnosis is chronic heart failure, while factors associated with ACS without TC include non-white races, prior CABG, PCI, myocardial infarction, ICD/PPM, stroke, hypertension, hyperlipidemia, diabetes, obesity, valvular heart disease, atrial fibrillation and renal failure. Increased risk of TC was seen with the increase in the median household incomes by zip code, with the 76th–100th percentile group having 16% higher odds of developing TC as compared with the 0–25th percentile cohort.

**Table 2:** Predictors of Takotsubo cardiomyopathy in adult inpatients with acute coronary syndrome

Variables	OR (95% CI)	P value
Age (per 10 years)	0.92 (0.91 to 0.93)	**<0.001**
Female	6.78 (6.47 to 7.09)	**<0.001**
Race		
White	Reference	
Black	0.47 (0.44 to 0.50)	**<0.001**
Hispanic	0.70 (0.65 to 0.75)	**<0.001**
Asian or Pacific Islander	0.76 (0.71 to 0.81)	**<0.001**
Hospital region		
Northeast	Reference	
Midwest	1.22 (1.14 to 1.29)	**<0.001**
South	1.08 (1.02 to 1.15)	**0.004**
West	1.26 (1.18 to 1.34)	**<0.001**
Hospital bed size		
Small	Reference	
Medium	1.13 (1.07 to 1.20)	**<0.001**
Large	1.34 (1.27 to 1.41)	**<0.001**
Teaching hospital		
Rural	Reference	
Urban, non-teaching	1.34 (1.23 to 1.47)	**<0.001**
Urban, teaching	1.68 (1.54 to 1.82)	**<0.001**
Median household income by zip code		
0–25th percentile	Reference	
26th–50th percentile	1.06 (1.01 to 1.11)	**0.013**
51st–75th percentile	1.15 (1.10 to 1.21)	**<0.001**
76th–100th percentile	1.16 (1.10 to 1.23)	**<0.001**
Past medical history		
Old myocardial infarction	0.86 (0.81 to 0.91)	**<0.001**
History of CABG	0.32 (0.29 to 0.36)	**<0.001**
History of PCI	0.98 (0.95 to 1.02)	0.467
History of ICD/PPM	0.64 (0.58 to 0.70)	**<0.001**
History of stroke	0.81 (0.77 to 0.86)	**<0.001**
Cardiac arrhythmias	0.99 (0.94 to 1.04)	0.800
Atrial fibrillation	0.68 (0.64 to 0.72)	**<0.001**
Valvular heart disease	0.66 (0.62 to 0.69)	**<0.001**
Hypertension	0.63 (0.60 to 0.65)	**<0.001**
Hyperlipidemia	0.97 (0.94 to 1.01)	**<0.001**
Diabetes mellitus	0.65 (0.63 to 0.68)	**<0.001**
Chronic heart failure	1.60 (1.54 to 1.66)	**<0.001**
Renal failure	0.48 (0.46 to 0.51)	**<0.001**
Obesity	0.61 (0.58 to 0.64)	**<0.001**
Smoking	1.02 (0.99 to 1.06)	0.112

CABG, coronary artery bypass grafting; ICD/PPMimplantable cardiac defibrillator/permanent pacemakerPCI, percutaneous coronary intervention

### Association with adverse outcomes and novel risk model

Multivariable analysis predictors for in-hospital mortality and morbidity outcomes among patients with TC are listed in table 3. Older age, male sex, non-white race, prior cardiac arrhythmias, diabetes, cardiogenic shock and vasopressor use were independently associated with higher risk of all four adverse outcomes. Cardiogenic shock was the strongest predictor with OR (95% CI) of 4.13 (3.62 to 4.72) for all-cause mortality, 3.23 (2.74 to 3.80), p<0.001 for cardiovascular mortality and vasopressor was the strongest predictor with 2.95 (2.61 to 3.34) for AKI and stroke 1.52 (1.23 to 1.88), p<0.001. The 26th–50th percentile cohort was associated with higher rates of in-hospital all-cause (1.15 (1.01 to 1.30), p=0.027) and cardiovascular mortality (1.26 (1.06 to 1.49), p=0.006) in TC.

**Table 3:** Multivariable analyses of in-hospital mortality and complications in adult patients with Takotsubo cardiomyopathy

Variables	In-hospital mortality		Cardiovascular mortality		Acute kidney injury		Stroke	
	OR (95% CI)	P value	OR (95% CI)	P value	OR (95% CI)	P value	OR (95% CI)	P value
Age (per 10 years)	1.19 (1.16 to 1.24)	**<0.001**	1.13 (1.08 to 1.19)	**<0.001**	1.02 (1.00 to 1.04)	**0.007**	1.04 (1.00 to 1.08)	**0.032**
Female	0.54 (0.49 to 0.60)	**<0.001**	0.63 (0.55 to 0.72)	**<0.001**	0.50 (0.47 to 0.53)	**<0.001**	0.66 (0.58 to 0.76)	**<0.001**
White	Reference	Reference	Reference	Reference	Reference	Reference	Reference	Reference
Black	1.23 (1.05 to 1.43)	**0.007**	1.19 (0.97 to 1.46)	0.090	1.32 (1.19 to 1.45)	**<0.001**	1.33 (1.11 to 1.58)	**0.002**
Hispanic	1.24 (1.04 to 1.47)	**0.012**	1.08 (0.85 to 1.38)	0.500	0.96 (0.86 to 1.08)	0.599	1.13 (0.92 to 1.38)	0.218
Asian or Pacific Islander	1.43 (1.24 to 1.65)	**<0.001**	1.30 (1.07 to 1.57)	**0.007**	1.12 (1.01 to 1.23)	**0.019**	1.22 (1.03 to 1.45)	**0.021**
Hospital region								
Northeast	Reference	Reference	Reference	Reference	Reference	Reference	Reference	Reference
Midwest	0.98 (0.86 to 1.13)	0.877	1.04 (0.87 to 1.25)	0.606	0.97 (0.89 to 1.05)	0.471	1.05 (0.90 to 1.24)	0.483
South	0.90 (0.79 to 1.03)	0.152	1.02 (0.85 to 1.21)	0.808	0.88 (0.81 to 0.96)	**0.003**	1.09 (0.94 to 1.27)	0.221
West	0.96 (0.83 to 1.11)	0.626	1.08 (0.90 to 1.29)	0.385	0.86 (0.79 to 0.94)	**0.001**	1.07 (0.91 to 1.26)	0.359
Hospital bed size								
Small	Reference	Reference	Reference	Reference	Reference	Reference	Reference	Reference
Medium	1.10 (0.94 to 1.28)	0.200	1.01 (0.83 to 1.24)	0.858	1.04 (0.96 to 1.13)	0.304	1.26 (1.05 to 1.51)	**0.010**
Large	1.41 (1.23 to 1.61)	**<0.001**	1.27 (1.06 to 1.51)	**0.007**	1.04 (0.97 to 1.12)	0.218	1.69 (1.44 to 1.98)	**<0.001**
Teaching hospital								
Rural	Reference	Reference	Reference	Reference	Reference	Reference	Reference	Reference
Urban, non-teaching	1.13 (0.90 to 1.43)	0.261	1.42 (1.04 to 1.95)	**0.027**	1.14 (0.99 to 1.30)	0.053	1.12 (0.83 to 1.49)	0.445
Urban, teaching	1.36 (1.10 to 1.67)	**0.004**	1.43 (1.07 to 1.91)	**0.015**	1.31 (1.16 to 1.49)	**<0.001**	1.77 (1.36 to 2.30)	**<0.001**
Median household income by zip code								
0–25th percentile	Reference	Reference	Reference	Reference	Reference	Reference	Reference	Reference
26th–50th percentile	1.15 (1.01 to 1.30)	**0.027**	1.26 (1.06 to 1.49)	**0.006**	0.99 (0.93 to 1.07)	0.986	0.97 (0.85 to 1.12)	0.771
51st–75th percentile	1.06 (0.93 to 1.20)	0.356	1.04 (0.87 to 1.24)	0.623	0.98 (0.91 to 1.06)	0.700	0.97 (0.84 to 1.12)	0.698
76th–100th percentile	1.00 (0.87 to 1.14)	0.970	1.00 (0.84 to 1.20)	0.935	0.94 (0.87 to 1.02)	0.146	0.89 (0.76 to 1.04)	0.166
Old MI	0.75 (0.63 to 0.88)	**0.001**	0.78 (0.61 to 0.98)	**0.037**	0.78 (0.71 to 0.85)	**<0.001**	0.86 (0.73 to 1.02)	0.099
History of CABG	1.80 (1.34 to 2.42)	**<0.001**	1.95 (1.35 to 2.83)	**<0.001**	0.89 (0.72 to 1.09)	0.265	1.16 (0.81 to 1.66)	0.396
History of PCI	0.61 (0.55 to 0.68)	**<0.001**	0.69 (0.60 to 0.79)	**<0.001**	0.79 (0.75 to 0.84)	**<0.001**	0.82 (0.74 to 0.92)	**0.001**
History of ICD/PPM	0.71 (0.55 to 0.91)	**0.001**	0.66 (0.48 to 0.92)	**0.014**	0.77 (0.67 to 0.88)	**<0.001**	0.58 (0.42 to 0.81)	**0.001**
History of stroke	0.93 (0.79 to 1.08)	0.381	0.78 (0.62 to 0.98)	**0.034**	0.89 (0.81 to 0.98)	**0.018**	1.97 (1.71 to 2.27)	**<0.001**
Cardiac arrhythmias	1.67 (1.48 to 1.87)	**<0.001**	2.95 (2.55 to 3.42)	**<0.001**	1.23 (1.14 to 1.32)	**<0.001**	1.13 (0.98 to 1.31)	0.081
Atrial fibrillation	0.98 (0.86 to 1.12)	0.798	0.63 (0.54 to 0.74)	**<0.001**	1.08 (0.99 to 1.17)	0.073	1.21 (1.03 to 1.43)	**0.020**
Valvular heart disease	0.74 (0.64 to 0.85)	**<0.001**	0.70 (0.58 to 0.85)	**<0.001**	0.94 (0.86 to 1.02)	0.143	0.98 (0.83 to 1.15)	0.821
Hypertension	0.75 (0.68 to 0.83)	**<0.001**	0.78 (0.68 to 0.89)	**<0.001**	0.97 (0.91 to 1.03)	0.388	0.99 (0.88 to 1.12)	0.996
Hyperlipidemia	0.54 (0.49 to 0.59)	**<0.001**	0.56 (0.49 to 0.65)	**<0.001**	0.73 (0.69 to 0.77)	**<0.001**	1.06 (0.95 to 1.18)	0.238
Diabetes mellitus	1.13 (1.02 to 1.26)	**0.016**	1.08 (0.93 to 1.24)	0.288	1.35 (1.27 to 1.44)	**<0.001**	1.26 (1.12 to 1.41)	**<0.001**
Chronic heart failure	0.90 (0.82 to 0.98)	**0.028**	2.50 (2.20 to 2.85)	**<0.001**	1.69 (1.60 to 1.78)	**<0.001**	1.05 (0.94 to 1.17)	0.357
Chronic renal failure	1.33 (1.18 to 1.50)	**<0.001**	1.43 (1.23 to 1.66)	**<0.001**	4.65 (4.34 to 4.98)	**<0.001**	0.87 (0.75 to 1.00)	0.065
Obesity	0.91 (0.79 to 1.04)	0.194	0.96 (0.79 to 1.15)	0.676	1.08 (0.99 to 1.17)	0.057	0.79 (0.66 to 0.93)	**0.007**
Smoking	0.74 (0.67 to 0.81)	**<0.001**	0.69 (0.61 to 0.79)	**<0.001**	0.72 (0.69 to 0.77)	**<0.001**	0.73 (0.66 to 0.82)	**<0.001**
Cardiogenic shock	4.13 (3.62 to 4.72)	**<0.001**	3.23 (2.74 to 3.80)	**<0.001**	2.70 (2.44 to 2.99)	**<0.001**	1.29 (1.06 to 1.56)	**0.009**
Balloon pump	1.33 (1.02 to 1.74)	**0.033**	1.51 (1.11 to 2.06)	**0.009**	1.21 (0.97 to 1.51)	0.081	1.05 (0.70 to 1.57)	0.812
Vasopressor use	3.81 (3.28 to 4.43)	**<0.001**	3.06 (2.55 to 3.68)	**<0.001**	2.95 (2.61 to 3.34)	**<0.001**	1.52 (1.23 to 1.88)	**<0.001**

CABG, coronary artery bypass grafting; ICD/PPMimplantable cardiac defibrillator/permanent pacemakerMI, myocardial infarctionPCI, percutaneous coronary intervention

**Figure 1:**
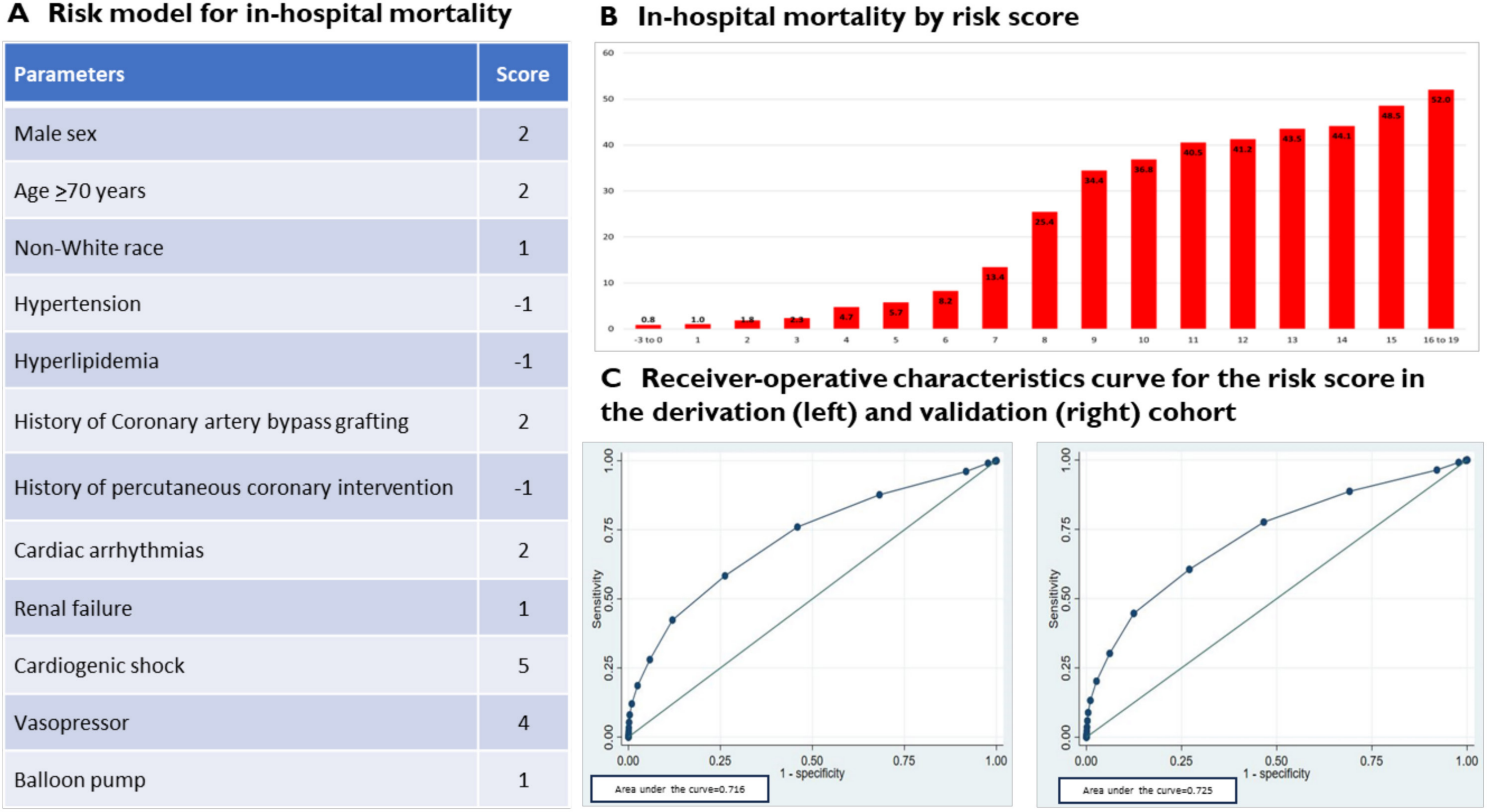
Risk prediction model for in-hospital mortality in Takotsubo cardiomyopathy and in-hospital mortality rate by score.

Based on the multivariable analysis results, a newly developed risk score for predicting in-hospital mortality in patients with TC was created and is presented in the figure 1. The score spans from a minimum of −3 to a maximum of 15, categorised into low (−3 to +3), medium (4-7) and high scores (8-19), with the parameters of male sex, age >70 years, non-white race, hypertension, hyperlipidemia, history of CABG, history of PCI, cardiac arrhythmias, renal failure, vasopressor use and cardiogenic shock. This score and the in-hospital mortality rates for TC by this risk score are illustrated in the figure 1, which steadily increases as the score increases. The overall AUC for this score to discriminate in-hospital mortality in our TC cohort was 0.718 (95% CI 0.718 to 0.719). The Brier score was 0.05, and the R^2^ index was 0.12 via bootstrapping. The link to the calculator is https://takotsubocardiomyopathymortalityscore-calculator.web.val.run/.

## Discussion

This national sample study over a 5-year period of patients admitted with TC compared with other patients with ACS had several important findings. TC was diagnosed in 2.8% of ACS admissions, and unique and differing demographics, comorbidities and presentation characteristics of TC compared with other ACS were identified. The in-hospital all-cause mortality rate was comparable but slightly lower for TC than other ACS (7.0% vs 8.0%). Cardiovascular mortality was similar in TC and ACS, while stroke was higher and AKI was lower. Factors independently associated with TC among patients with ACS and in-hospital outcomes in patients with TC were analysed. Based on this, a novel risk score for predicting in-hospital all-cause mortality in patients with TC was developed, with higher internal validity performance based on the high AUC.

### Characteristics and predictors of Takotsubo cardiomyopathy

The key clinical characteristics of patients with TC we identified compared with other patients with ACS include a higher proportion 82.5% in females, a lower proportion of non-white race, a lower prevalence of most cardiovascular diseases and risk factors while depression was more common, and interestingly, higher proportion presenting with cardiogenic shock and vasopressor use but less balloon pump use. Female predominance in patients with TC is well documented in the literature.^8^,^14^ Non-white races making up a lower proportion of TC than other patients with ACS have been previously reported.^15^ On the other hand, prior studies have shown older age (>55 years of age) had around 5 times higher odds of having TC as compared with their younger counterparts, especially in women.^15^ Similarly, older age is associated with a higher risk of other ACS, and the mean age of both groups (68–69 years) was similar in our study and prior studies.^16^,^18^ This is important to note as postmenopausal women are more likely to get TC and also have a high prevalence of many cardiovascular risk factors compared with younger women.^1^

As expected, most cardiovascular diseases and risk factors are more common in ACS than in patients with TC. Consistent with prior reports and in multivariable analysis, chronic heart failure was independently associated with TC in this study, suggesting that underlying diseased myocardial tissue and function predispose to the acute ventricular dysfunction seen in TC. Prior studies have also shown a strong association between heart failure and TC, which coincides with our findings.^9 19^ Further studies are necessary to investigate if chronic heart failure is associated with a specific pattern of TC, which is most commonly apical, but other midventricular, basal and focal subtypes have been reported,^19^ as well as a lower chance of reversibility of LV dysfunction. Cardiogenic shock was more common in the presentation of TC compared with other patients with ACS, which may be associated with all TC presenting with LV dysfunction compared with only some patients with ACS, so TC is more likely to have severe heart failure symptoms.

### Outcomes and novel risk model for Takotsubo cardiomyopathy

The in-hospital mortality rate in patients with TC with ACS in our study was 7%, similar to rates reported in previous studies in patients with TC (1%–8%).^520^,^24^ Cardiovascular mortality rates were similar at 4.0%, suggesting non-cardiovascular rates were also marginally lower for TC than other ACS. Stroke rates were higher for TC, which may be associated with a higher proportion of patients with TC with LV (especially apical) dysfunction, precipitating thrombus formation and cardioembolic stroke. Other studies have reported approximately 40% of patients with TC with cardioembolic events having LV thrombus,^25 26^ and TC being associated with both atrial fibrillation and stroke.^27^ AKI was more common in patients with ACS compared with those with TC. This may be related to the higher prevalence of cardiovascular risk factors and diseases in the ACS population. as well as the greater contrast load typically administered during PCI that is often necessary for other forms of ACS but not for TC.

We also reported the key adverse predictors of both in-hospital mortality and morbidities in patients with TC. Older age, male sex, non-white race, prior cardiac arrhythmias, diabetes, vasopressor use and cardiogenic shock were independently associated with higher risk of all four adverse outcomes. Older age is an established adverse prognosticator in TC,^28^ and importantly, the male sex has also been reported in several other TC studies as having a higher risk of mortality than women.^1416 29^,^36^ Sex differences in TC mortality are poorly understood, and one observation is that men with TC have more underlying comorbidities and more severe illness and physical as opposed to emotional stress triggers than women with TC.^9 37^ Non-white race having a higher risk of adverse outcomes was also previously reported.^38 39^ The precise cause of racial disparities in outcomes has not been thoroughly defined in the literature. However, hypotheses suggest that genetic variances affecting sympathetic activity regulation and distinctions in endothelial hemostasis may contribute to these differences.^40^,^44^ Not unexpectedly, cardiogenic shock was the strongest predictor of adverse in-hospital mortality, given its likelihood to lead to severe cardiopulmonary and multiorgan compromise.^16^ Furthermore, the use of vasopressors and their association with adverse outcomes may be a consequence of cardiogenic shock and stem from their potential to exacerbate LV outflow tract obstruction, which in turn may precipitate cardiogenic shock. Based on these analyses, we developed, to our knowledge, the first TC-specific risk score to predict in-hospital mortality, aimed at facilitating the clinical management of patients with TC. This risk score demonstrated moderate performance, with an area under the curve of 0.718 in this cohort.

### Clinical implications

There are several clinical implications for our study. Among patients with ACS presenting with clinical signs, ECG changes and abnormal cardiac troponin levels, TC remains a challenging diagnosis. It requires cardiac imaging to document acute LV dysfunction—often regional—and to assess for possible LV thrombus formation and dynamic LV outflow tract obstruction, which frequently resolves on follow-up imaging. Additionally, coronary catheterisation is necessary to rule out a culprit obstructive coronary lesion, although coronary artery disease may be concomitantly present.^1 4^ A position statement from the European Society of Cardiology recommended oral anticoagulant therapy for at least 3 months in patients with TC with LV thrombus. Prophylactic oral anticoagulation can be considered in patients with high-risk TC.^45^ Results from a German Registry study showed that LV thrombus can occur in 2.2% of cases of TC in the acute phase, which can lead to a cerebrovascular accident in 17% of cases.^46^ After meticulous clinical and echocardiographic evidence of LV thrombus resolution and LV function recovery, oral anticoagulation (vitamin K antagonists) was discontinued after 3 months with no reports of stroke postdiscontinuation.^46^ Although there are no concrete guidelines on the use of novel oral anticoagulants in patients with TC, they can still be considered a reasonable option for stroke prevention.^47^ The factors we identified as associated with TC may help distinguish it from other forms of ACS when cardiac tests are inconclusive or when catheterisation is contraindicated. More importantly, higher risk scores from the presence of multiple adverse prognosticators should alert clinicians to allocate more healthcare resources and ensure careful management of these patients. They may require more intensive monitoring and/or specialised interventions while also providing patients and families with prognostic information about potential disease trajectories and outcomes. Along similar lines, the InterTAK score was established, which estimated the TC presence and differentiated TC from ACS with high sensitivity and specificity, respectively.^48^ Lower-risk patients may only need standard heart failure medical therapy. Current management of TC is primarily based on guidelines for treating acute and chronic heart failure. Acute management may include diuretics, oxygen therapy and treatment for cardiogenic shock treatment if present. In the medium term, the focus shifts to goal-directed medical therapy, which typically includes beta-blockers and renin-angiotensin-aldosterone system blockers.^49 50^ Further research remains necessary to assess how this risk model can guide the management of patients in different risk categories and whether this strategy can improve TC clinical outcomes.

### Limitations

This study has some limitations. It is a retrospective observational cohort study, which carries intrinsic biases, although it is one of the largest multicentre studies on TC in the literature. NIS is an administrative discharge-level national database, so diagnostic codes are prone to inaccuracies and misclassification for TC, clinical characteristics and outcomes. An important weakness of the NIS dataset is the lack of laboratory imaging (including echocardiography, cardiac magnetic resonance and coronary angiography) and treatments (including medications, interventions and surgery). Therefore, these variables could not be analysed for their associations with TC diagnosis and prognosis or incorporated into risk models. Another limitation of our analysis is the potential for the table 2 fallacy, which arises when interpreting associations between covariates and outcomes without adequately adjusting for the primary exposure of interest. While we reported the crude associations between various patient characteristics and outcomes in table 3, these associations may be misleading if interpreted without considering the influence of the primary exposure or other confounders. Specifically, the relationships observed between covariates and outcomes could reflect confounding rather than true causal relationships. To mitigate this limitation, we adjusted for the main exposure and relevant confounders in our multivariable model. However, readers should be cautious in interpreting the unadjusted associations presented in table 3, as they do not fully account for the complex interplay between the primary exposure, covariates and outcomes. Lastly, long-term follow-up outcomes beyond index hospitalisation are not available.

## Conclusion

TC remains a challenging diagnosis that makes up a significant minority of ACS. Unique characteristics and factors associated with TC among patients with ACS were identified. TC had a slightly lower but significant in-hospital mortality rate at 7.0% compared with other ACS at 8.0%, with similar cardiovascular mortality, higher stroke and lower AKI rate. Independent predictors of in-hospital mortality and morbidities in patients with TC were analysed, from which a risk model for in-hospital mortality was developed, with moderate discriminative ability in both the derivation and validation cohorts. Despite the optimal management strategy of TC being controversial, our novel scores help to stratify patients with TC into different risk categories to potentially aid management. Further research is necessary to externally assess this score and evaluate whether applying this score to guide management improves the clinical outcomes of patients with TC.

## supplementary material

10.1136/openhrt-2024-002922 online supplemental file 1

## Data Availability

All data relevant to the study are included in the article or uploaded as supplementary information.
