# Supplementary material for: Contemporary characteristics, outcomes and novel risk score for Takotsubo cardiomyopathy: a national inpatient sample analysis
Source: Open Heart. 2024 Oct 7;11(2):e002922. doi: 10.1136/openhrt-2024-002922 (PMC11459304; doi:10.1136/openhrt-2024-002922)
Supplement: online supplemental file 1 [file openhrt-11-2-s001.pdf]

Supplementary materials

Table S1: ICD-10 codes for the variables

**Variables**	**ICD-10 codes**
Takotsubo cardiomyopathy	I5181
Coronary artery disease	I201, I25, I208, I209
Chronic heart failure	I5022, I5032, I5042, I509
Prior ICD/PPM	Z4502, Z95810, Z950, Z4501, Z45010 Z45018
Old myocardial infarction	I252
History of PCI	Z9861, I2510
History of CABG	Z951
Acute stroke	I6781, I6782, I63, G46, I69
Diabetes mellitus	E08, E09, E10, E11, E13
Hypertension	I1
Hyperlipidemia	E780, E781, E782, E783, E784, E785
Obesity	E660, E661, E662, E668, E669, Z683, Z684, O9921
Cannabis use	F12
Opioids use	F11
Cocaine	F14
Sedative/hypnotics	F13
Stimulant use	F15
Smoking	F17, Z720, Z87891
Anxiety	F41
Cardiogenic shock	R570
ECMO	5A15
LVAD	02HA0QZ, 02HA2QZ, 02HA4QZ
Ballon pump	5A02
Vasopressor use	3E030XZ, 3E030XZ, 3E040XZ, 3E043XZ, 3E053XZ, 3E060XZ, 3E063XZ, 3E050XZ
Cardiac arrest	I462, I468, I469
Acute kidney injury	N17
Atrial fibrillation	I48
Cardiac tamponade	I314
Acute mitral regurgitation	I341, I340, I349

Abbreviations: ICD-10: International Classification of Disease, Tenth Revision; PCI: Percutaneous coronary intervention; CABG: Coronary artery bypass grafting; ICD/PPM: Implantable cardioverter defibrillator/permanent pacemaker; ECMO: Extracorporeal membrane oxygenation; LVAD: Left ventricle assist device

Figure S1: Trend of Takotsubo cardiomyopathy by year. P-value based on Pearson's Chi-Square test (p=<0.001)

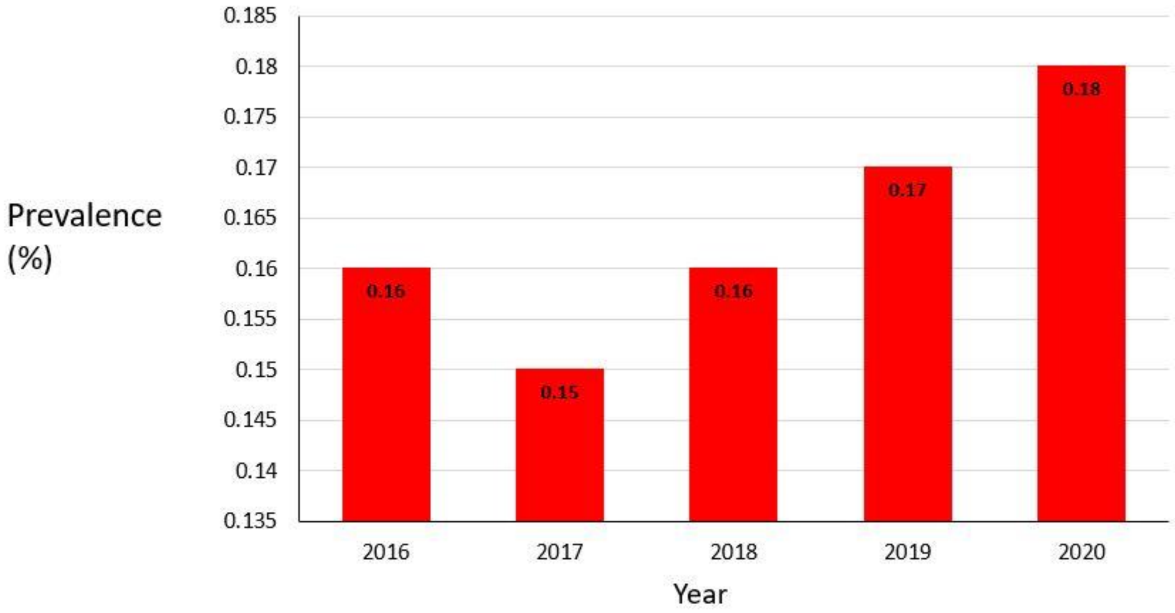

Figure S2: Seasonal variations in a) Takotsubo cardiomyopathy admissions and b) Takotsubo cardiomyopathy in-hospital mortality. P-values based on Pearson's chi-square test.

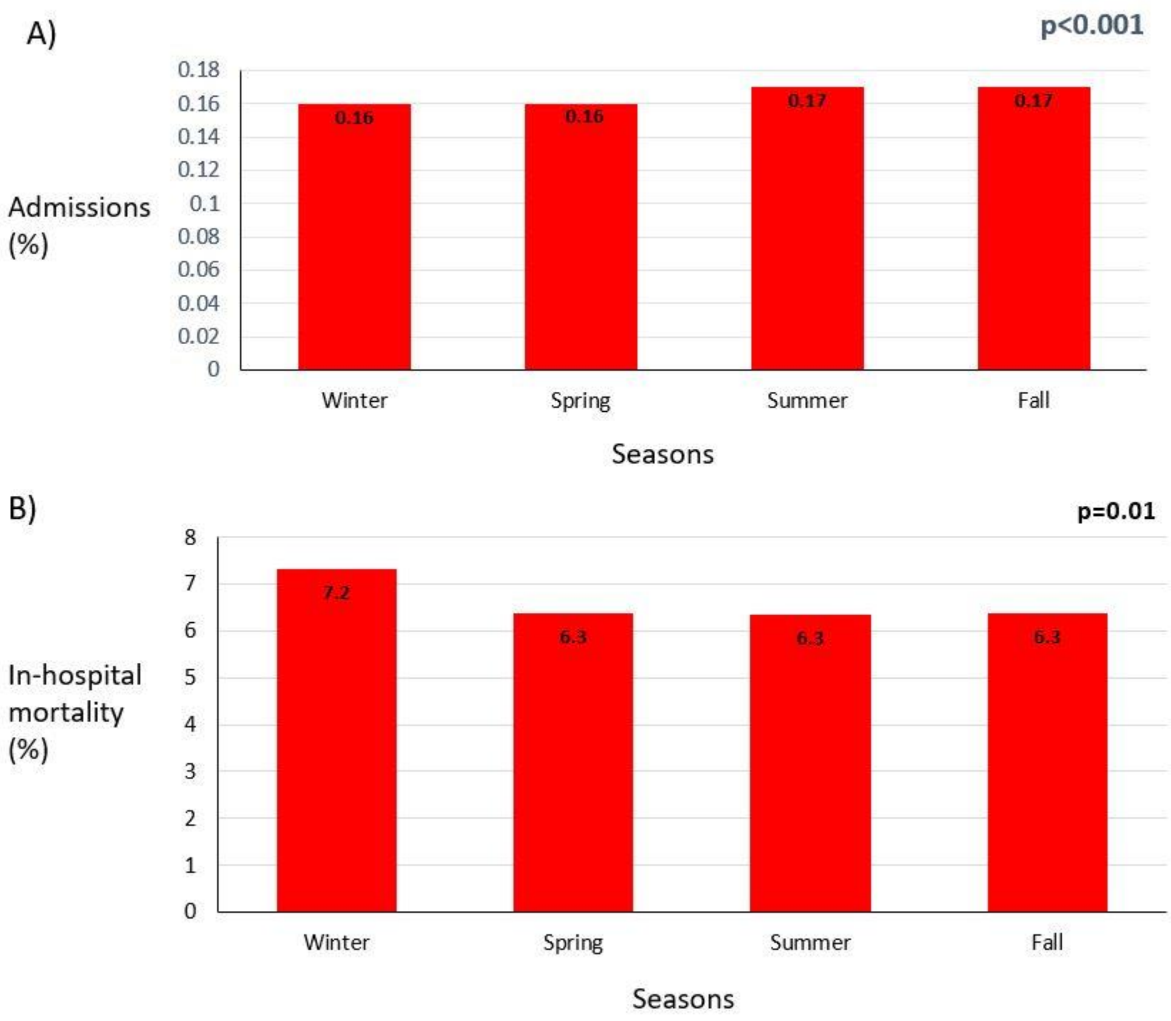
A)
p<0.001
Admissions (%)
0.18
0.16
0.14
0.12
0.1
0.08
0.06
0.04
0.02
0
0.16
0.16
0.17
0.17
Winter
Spring
Summer
Fall
Seasons
B)
p=0.01
In-hospital mortality (%)
8
7
6
5
4
3
2
1
0
7.2
6.3
6.3
6.3
Winter
Spring
Summer
Fall
Seasons
